# Comparison of metaheuristics to measure gene effects on phylogenetic supports and topologies

**DOI:** 10.1186/s12859-018-2172-8

**Published:** 2018-07-09

**Authors:** Régis Garnier, Christophe Guyeux, Jean-François Couchot, Michel Salomon, Bashar Al-Nuaimi, Bassam AlKindy

**Affiliations:** 1 FEMTO-ST Institute, UMR 6174 CNRS, DISC Computer Science Department, Univ. Bourgogne Franche-Comté (UBFC), 16 Route de Gray, Besançon, 25000 France; 2 Department of Computer Science, Mustansiriyah University, Baghdad, 10052 Iraq; 3 Department of Computer Science, Diyala University, Diyala, 32001 Iraq

**Keywords:** Chloroplasts, Phylogeny, Metaheuristics, Genetic algorithms, Lasso test, Binary particle swarm optimization, Simulated annealing

## Abstract

**Background:**

A huge and continuous increase in the number of completely sequenced chloroplast genomes, available for evolutionary and functional studies in plants, has been observed during the past years. Consequently, it appears possible to build large-scale phylogenetic trees of plant species. However, building such a tree that is well-supported can be a difficult task, even when a subset of close plant species is considered. Usually, the difficulty raises from a few core genes disturbing the phylogenetic information, due for example from problems of homoplasy. Fortunately, a reliable phylogenetic tree can be obtained once these problematic genes are identified and removed from the analysis.Therefore, in this paper we address the problem of finding the largest subset of core genomes which allows to build the best supported tree.

**Results:**

As an exhaustive study of all core genes combination is untractable in practice, since the combinatorics of the situation made it computationally infeasible, we investigate three well-known metaheuristics to solve this optimization problem. More precisely, we design and compare distributed approaches using genetic algorithm, particle swarm optimization, and simulated annealing. The latter approach is a new contribution and therefore is described in details, whereas the two former ones have been already studied in previous works. They have been designed *de novo* in a new platform, and new experiments have been achieved on a larger set of chloroplasts, to compare together these three metaheuristics.

**Conclusions:**

The ways genes affect both tree topology and supports are assessed using statistical tools like Lasso or dummy logistic regression, in an hybrid approach of the genetic algorithm. By doing so, we are able to provide the most supported trees based on the largest subsets of core genes.

## Background

These last years the investigation of the evolutionary relationship between different plants has benefited from the multiplication of the available chloroplast sequences. Indeed, thanks to various tools it is possible to process these sequences in order to build a phylogenetic tree that accurately characterizes the evolutionary lineages among the chloroplasts. Efficient coding sequence prediction and annotation tools have been developed to deal specifically with chloroplasts, for example DOGMA [1], and there is also a great choice for the alignment of sequences. Moreover, given a set of sequences or characters, many well-established bioinformatics programs based on Bayesian inference or maximum likelihood, like BEAST or RAxML [2], can be used to reconstruct a phylogenetic tree. The objective is usually to obtain a reliable phylogeny on which biological investigations can be applied. For instance, in, a comparative analysis of expressed rice gene homologues in 48 other diverse eukaryotic species has been performed, and a phylogenetic tree of life based on 98 of these genes conserved across the species has been computed using such software. It has been used to estimate more accurately the divergence time among a large number of species pairs. However, the genome of rice plant has approximately 40,000 genes, and their tree has been computed using less than 1% of such genes.

Several methods can be used to estimate the robustness of the produced tree, the most widely used are the bootstrap and the decay (or Bremer) analyses [3, 4]. Obviously, a first condition to be able to build a phylogenetic tree for a given set of close plant species is to identify as precisely as possible the corresponding core genome [5] (the set of genes in common). However, even if the core genome is large and accurate, the resulting phylogeny is not necessarily well-supported [6]. In fact, the core genome genes are not constrained through evolution in a similar way. On the one hand some evolve under strong evolutionary constraints and thus reflect the story of the species while, on the other hand, other genes evolve more freely due to a lower role in the survival and adaptability of a species. The latter tell their own history and thus disturb the phylogenetic information. Furthermore, the way the robustness and accuracy of the obtained phylogenetic tree are altered by the amount of used data for the reconstruction process is not completed understood. Nevertheless, if we consider a set of species reduced to lists of gene sequences, an obvious dependence between the chosen subset of sequences and the obtained tree (topology, branch length, and/or robustness) can be observed. This dependence is usually regarded by the mean of gene trees merged in a phylogenetic network. In fact, phylogenetic networks are necessary to represent events like horizontal gene transfers, but statistical methods to infer such networks are still limited and under development.

In this article, we consider the situation from a dual point of view, that consists in starting with the complete core genome and then to remove the genes responsible for inconsistent phylogenetic signal. In other words, the objective is to find the largest part of the core genome that produces a phylogenetic tree as supported as possible, and which therefore gives the fairest view of the relationships between most of the sequences under consideration. Searching the problematic genes by exhaustively testing the combinations of core genome genes is nonsense due their huge number. Therefore, to speed up the finding of a satisfactory combination we rather consider metaheuristics. The first one, introduced in a previous work [7], is an ad hoc Genetic Algorithm (GA) which in some cases is not able to converge towards a suitable solution. Next, a Binary Particle Swarm Optimization (PSO) approach has been published in the the CIBB proceedings book [8]. Finally, in this article, which extends and improves the two former ones, we study the relevance of the Simulated Annealing (SA) algorithm to fulfill the optimization task. Also notice that the different metaheuristics have been executed in a distributed manner using supercomputing facilities. To sum up, the contribution of this article is threefold: first, it proposes a new simulated annealing approach, second a new version of the PSO, and third a comparison of the three metaheuristics on a large number of new groups of species. Compared to usual articles studying the tree of life like, our approach is diametrically opposed: instead of using existing phylogenetic software on a small collection of core genes, in order to provide new discoveries on some aspects of the Evolution, we propose a pipeline of 3 metaheuristics, to find the largest subset of core genes leading to the most supported tree.

## Methods

### Problem description

Let us introduce the problem of determining a phylogeny (evolution tree) for a given set of species by considering a set of chloroplast genomes that have been annotated using DOGMA [1] (the approach we applied is detailed in “Results” section). To start we need to pick one or several genes on which the phylogeny will be based. Therefore we use the restricted core genome [9, 10], which consists of conserved genes present everywhere, whose size is larger than one hundred genes when the species are close enough. Then multiple sequence alignments are performed using muscle [11] and finally a phylogenetic tree is inferred thanks to the maximum-likelihood tree builder RAxML [2].

The relevance of the obtained tree is then assessed by its bootstrap values: if these ones are all above 95 the tree is well-supported, in which case we can reasonably estimate that the phylogeny of these species is solved. Bootstrapping is a random sampling technique commonly used to estimate the significance of branches of a phylogenetic tree. It consists to randomly select columns in the aligned DNA core sequences to be neglected during the tree building process and to check whether the same nodes are recovered. A large number of bootstrap repetitions, usually between 50 and 1000, are used to assess the tree reliability. As an illustration, a node which appears 95 times out of 100 by dropping a column means that the node is well-supported. Conversely, a low support value claims that a reduced part of the alignment supports the node, since by removing columns the node is reconstructed in different ways.

When such a well-supported tree is not built, but rather a tree having some branches exhibiting low supports, some genes of the core genome can be responsible of this lack of support. The objective is then to identify the most supported tree using the largest subset of core genes, a typical optimization problem. Obviously, the optimization problem we face cannot be solved by a brute force approach checking all possible combination of genes, due to the resulting combinatorial explosion. Indeed, for a core genome of *n* genes there would be 2^*n*^ trees to infer and that is clearly intractable in practice. To overcome such a combinatorial situation, a typical choice is to use a metaheuristic method.

In [7], we have first investigated the mixing of a genetic algorithm with Lasso tests to find problematic genes. Unfortunately, thorough and careful experimental investigations have led to results, recalled in Table 1, showing that this proposal is not able to predict the phylogeny of some particular plant orders. As can be seen, the lowest bootstrap value (or bootstrap score) obtained for 15 group of species is below 95 (column *b* in the table). The relevance of binary particle swarm optimization to find the largest subset of core genes has been studied in [8], producing slightly better bootstrap scores than GA with Lasso. In this paper we introduce a third well-known metaheuristic method, namely simulated annealing, and we compare the three approaches considering new sets of species. Like the two former ones, the computations with SA algorithm will be done in a distributed manner. Multiple algorithm instances will be launched using a same cooling schedule and at the end of each Markov chain, for a same temperature, a centralized communication scheme will take place.

**Table 1:** Results of genetic algorithm approach on various families

Group	occ	*c*	# taxa	*b*	Terminus	Likelihood	Outgroup
*Gossypium_group_0*	85	84	12	26	1	-84187.03	*Theo_cacao*
*Ericales*	674	84	9	67	3	-86819.86	*Dauc_carota*
*Eucalyptus_group_1*	83	82	12	48	1	-62898.18	*Cory_gummifera*
*Caryophyllales*	75	74	10	52	1	-145296.95	*Goss_capitis-viridis*
*Brassicaceae_group_0*	78	77	13	64	1	-101056.76	*Cari_papaya*
*Orobanchaceae*	26	25	7	69	1	-19365.69	*Olea_maroccana*
*Eucalyptus_group_2*	87	86	11	71	1	-72840.23	*Stoc_quadrifida*
*Malpighiales*	422	78	10	96	3	-91014.86	*Mill_pinnata*
*Pinaceae_group_0*	76	75	6	80	1	-76813.22	*Juni_virginiana*
*Pinus*	80	79	11	80	1	-69688.94	*Pice_sitchensis*
*Bambusoideae*	83	81	11	80	3	-60431.89	*Oryz_nivara*
*Chlorophyta_group_0*	231	24	8	81	3	-22983.83	*Olea_europaea*
*Marchantiophyta*	65	64	5	82	1	-117881.12	*Pice_abies*
*Lamiales_group_0*	78	77	8	83	1	-109528.47	*Caps_annuum*
*Rosales*	81	80	10	88	1	-108449.4	*Glyc_soja*
*Eucalyptus_group_0*	2254	85	11	90	3	-57607.06	*Allo_ternata*
*Prasinophyceae*	39	43	4	97	1	-66458.26	*Oltm_viridis*
*Asparagales*	32	73	11	98	1	-88067.37	*Acor_americanus*
*Magnoliidae_group_0*	326	79	4	98	3	-85319.31	*Sacc_SP80-3280*
*Gossypium_group_1*	66	83	11	98	1	-81027.85	*Theo_cacao*
*Triticeae*	40	80	10	98	1	-72822.71	*Loli_perenne*
*Corymbia*	90	85	5	98	2	-65712.51	*Euca_salmonophloia*
*Moniliformopses*	60	59	13	100	1	-187044.23	*Prax_clematidea*
*Magnoliophyta_group_0*	31	81	7	100	1	-136306.99	*Taxu_mairei*
*Liliopsida_group_0*	31	73	7	100	1	-119953.04	*Drim_granadensis*
*basal_Magnoliophyta*	31	83	5	100	1	-117094.87	*Ascl_nivea*
*Araucariales*	31	89	5	100	1	-112285.58	*Taxu_mairei*
*Araceae*	31	75	6	100	1	-110245.74	*Arun_gigantea*
*Embryophyta_group_0*	31	77	4	100	1	-106803.89	*Stau_punctulatum*
*Cupressales*	87	78	11	100	2	-101871.03	*Podo_totara*
*Ranunculales*	31	71	5	100	1	-100882.34	*Cruc_wallichii*
*Saxifragales*	31	84	4	100	1	-100376.12	*Aral_undulata*
*Spermatophyta_group_0*	31	79	4	100	1	-94718.95	*Mars_crenata*
*Proteales*	31	85	4	100	1	-92357.77	*Trig_doichangensis*
*Poaceae_group_0*	31	74	5	100	1	-89665.65	*Typh_latifolia*
*Oleaceae*	36	82	6	100	1	-84357.82	*Boea_hygrometrica*
*Arecaceae*	31	79	4	100	1	-81649.52	*Aegi_geniculata*
*PACMAD_clade*	31	79	9	100	1	-80549.79	*Bamb_emeiensis*
*eudicotyledons_group_0*	31	73	4	100	1	-80237.7	*Eryc_pusilla*
*Poeae*	31	80	4	100	1	-78164.34	*Trit_aestivum*
*Trebouxiophyceae*	31	41	7	100	1	-77826.4	*Ostr_tauri*
*Myrtaceae_group_0*	31	80	5	100	1	-76080.59	*Oeno_glazioviana*
*Onagraceae*	31	81	5	100	1	-75131.08	*Euca_cloeziana*
*Geraniales*	31	33	6	100	1	-73472.77	*Ango_floribunda*
*Ehrhartoideae*	31	81	5	100	1	-72192.88	*Phyl_henonis*
*Picea*	31	85	4	100	1	-68947.4	*Pinu_massoniana*
*Streptophyta_group_0*	31	35	7	100	1	-68373.57	*Oedo_cardiacum*
*Gnetidae*	31	53	5	100	1	-61403.83	*Cusc_exaltata*
*Euglenozoa*	29	26	4	100	3	-8889.56	*Lath_sativus*

Occ provides the number of genomes within the group while taxa is for the number of species. c and b respectively correspond to the percentage of core genes and the lowest bootstrap of the solution produced by the GA, while Likelihood is the likelihood of the best tree. Finally, Terminus specifies at which stage the GA stopped, while Outgroup is the considered outgroup

**Fig. 1:**
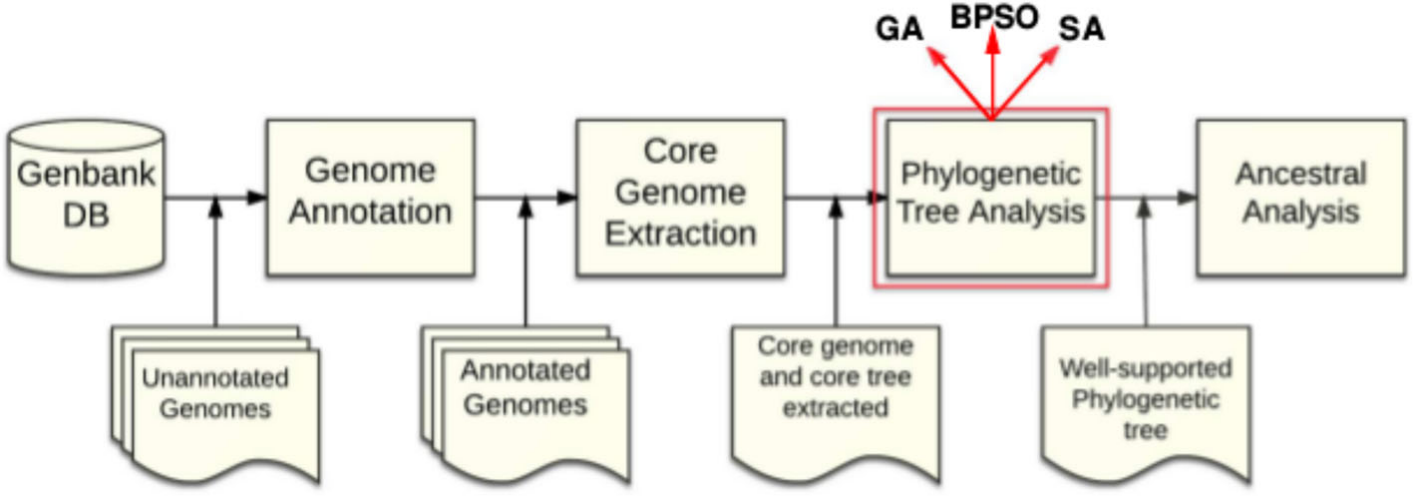
Overview of the proposed pipeline

To sum up, Fig. 1 gives an overview of the proposed pipeline to obtain the ancestral history of a set of species.

### Phylogenetic predictions using metaheuristics

#### Genetic algorithm approach

To make this article self-contained, we summarize hereafter the main steps of the genetic algorithm combined with Lasso test proposed in [7] aiming at finding problematic genes in core genome.

The *n* core genes are sorted alphabetically, and at each subset we associate a binary word of length *n*: its *i*-th character is 1 if and only if the *i*-th core gene is in the considered subset. In the proposed GA, a first stage to initialize the GA population (1) computes the set of *n*-length binary words containing the word having only 1’s (the whole core genome which is composed of *n* genes), (2) all words having exactly one 0 (all but 1 gene) further denoted as systematic mode, and (3) 200 words having between 2 and 10 0’s randomly located. Each of these words is associated with the score $\frac {b+p}{2}$ where *b* is the lowest bootstrap of the reconstructed phylogenetic tree and *p* is the percentage of considered core genes.

More precisely, the population is initialized with the 50 best words. Then, the GA iterates until discovering a word whose score is larger than 95, or at most for 200 iterations. Each iteration, which produces a new population, consists of the following steps:

1. Repeat 5 times a random pickup of a pair (*w*^1^,*w*^2^) of words and mix them using a crossover approach. In this step, indexes {1,…,*n*} are partitioned into *k*, $k \le \frac {n}{2}$, subsets *I*_1_,…*I*_*k*_. A new word *w* is then defined by $w_{i} = w_{i}^{1}$ if *i* belongs to some *I*_*j*_ where *j* is odd; otherwise $w_{i} = w_{i}^{2}$. The obtained words are added to the population *P*, resulting in population *P*_*c*_.
2. Mutate 5 words of the population *P*_*c*_. More precisely, for each of these words, *k* randomly selected binary values of *w* are switched leading to a new word. The mutated words are added to *P*_*c*_ leading to population *P*_*m*_.
3. Produce population *P*_*r*_ by adding 5 new random binary words having less than 10% of 0’s to *P*_*m*_.
4. Select the 50 best words in population *P*_*r*_ to form the new population *P*.

The aforementioned GA may not produce well-supported trees. Nevertheless, the whole set of produced words with their associated scores contains valuable information about which gene breaks supports. The idea is to focus on each topology having a frequency of occurrence larger than 10%. Then for each best word of these best topologies, and for each problematic bootstrap in its associated tree, we apply a Lasso test [12], which is recalled hereafter.

Let *W* be a *m*×*n* matrix where each line *W*_*i*_=(*X*_*i*1_,…,*X*_*ij*_,…,*X*_*in*_), 1≤*i*≤*m*, is a word. For each *W*_*i*_, let *Y*_*i*_ be the real positive support value for each problematic bootstrap *b* per topology and per gene. The Lasso test *β*=(*β*_1_,…,*β*_*i*_,…,*β*_*n*_) is thus defined by:

1 $$ {}\beta = \text{argmin} \left\{ \sum_{i=1}^{m} \left(Y_{i} - \sum\limits_{j=1}^{n} \beta_{j} X_{ij} \right)^{2} + \lambda\sum\limits_{j=1}^{n} | \beta_{j} | \right\}.  $$

It is not hard to see that the sign of *β*_*j*_ is positive (resp. negative) if the bootstrap support increases (resp. decreases) with respect to *j*.

This test allows thus to remove problematic genes, *i.e.*, genes *j*, 1≤*j*≤*n*, such that *β*_*j*_ is negative. Finally, a last genetic algorithm phase is launched on the updated population, in order to mix these promising words.

#### Binary particle swarm optimization approach

Particle Swarm Optimization [13] is a stochastic metaheuristic which has been successfully applied on artificial neural network training, fuzzy system control…In this scheme, an emergent behavior enables individual swarm members, particles, to take advantage from neighboring particles, which are closer numerically to the optimal solution. In the case of the standard Binary PSO (BPSO) model [14], each particle position is a *n*-length vector of binary values. A score function associates a real number to such kind of vector w.r.t. the optimization problem. BPSO aims at moving the particles in the *n* dimensions in order to obtain the optimal position with respect to this function.

More precisely, each particle *i* is represented by a *n*-length vector *X*_*i*_ of binary values, which has the same meaning than binary words in GA, indicating the gene contents of the associated core subset. Again, the *j*-th coordinate of *X*_*i*_ is 1 if and only if the associated *j*-th parameter is selected. A swarm of *L* particles is a list of position vectors (*X*_1_,*X*_2_,…,*X*_*L*_) together with their associated velocities (*V*_1_,*V*_2_,...,*V*_*L*_). Each *V*_*i*_ is a *n*-length vector of real numbers between 0 and 1. Each velocity vector *V*_*i*_ is updated as follows:

2 $$ {}V_{i}(t+1)= w V_{i}(t)+\phi_{1}\left(P_{i}^{\text{best}}-X_{i}\right)+\phi_{2}\left(P_{g}^{\text{best}}-X_{i}\right),  $$

where *w*, *ϕ*_1_, and *ϕ*_2_ are weighted parameters setting the level of each three trends for the particle, which are respectively to continue with the same inertia, to follow the direction of its own best neighbouring position $P_{i}^{\text {best}}$, or to follow the one of the global best known solution $P_{g}^{\text {best}}$.

Each position *X*_*ij*_ of the particle *i* is updated as follows:

3 $$\begin{array}{@{}rcl@{}} X_{ij}(t+1)= \left\{\begin{array}{ll} 1 & \text{if}\ {\tiny r}_{ij}\leq \frac{1}{1+e^{-V_{ij}(t+1)}}, \\ 0 & \text{otherwise}, \end{array}\right. \end{array} $$

where the scalar *r*_*ij*_ depends on both the particle *i* and the parameter *j*. It is not hard to recognize a choice guided by a threshold *r*_*ij*_ and a sigmoid [14] function. Let us now recall how this BPSO optimization scheme has been parameterized to solve our phylogeny problem [8].

Each vector *x*_*i*_∈{0,1}^*n*^ corresponds to a subset of core genes. It is associated with the deduced following data: the percentage *p* of considered core genes, the lowest bootstrap *b* of its induced phylogenetic tree, and, finally, the score $\frac {b+p}{2}$. The approach was to construct phylogenies based on neighbouring core genes: this leads to trees with similar topologies and with close bootstrap values with a high probability. During BPSO initialization, the *L* particles are randomly distributed among subsets of core genes (binary words) with a high percentage of 1’s. Further iterations move these particles in such a way that they will converge to an optimal node.

As in [15], at each iteration, the particle velocity is updated as in Eq. (2) where *ϕ*_1_ and *ϕ*_2_ are random numbers belonging to [0.1,0.5], and *w* linearly decreases between the first iteration and the last one from *w*_max_=0.9 to *w*_min_=0.4, as suggested in [16]. A large inertia weight indeed facilitates a global search, while a small inertia weight tends more to a local investigation.

A distributed version of BPSO algorithm has been proposed to minimize the execution time: each particle is executed in a worker core in order to compute its fitness value and obtained results are centralized by a supervisor master core. More precisely, the master initializes the particles of the swarm, distributes them to the workers, and waits until all of them have finished their task. It determines then the position of the particle that has the best fitness value as the global best position, and sends this information to the workers that update their respective particle velocity and position. This mechanism is repeated until a particle achieves a fitness value larger than or equal to 95 with a large set of included genes. In the following, two distributed versions of the BPSO are considered.

The former, further denoted as PSO version I, updates the velocity as follows:

4 $$ {}V_{i}(t+1)= x \cdot [V_{i}(t)+C_{1}(P_{i}^{best}-X_{i})+C_{2}(P_{g}^{best}-X_{i})]  $$

where *x*, *C*_1_, and *C*_2_ are weighted parameters setting the level of each three trends for the particle. The default values of these parameters are *C*_1_=*C*_2_=2.05, while *x*, which represents the constriction coefficient, is computed according to formula [17, 18]:

5 $$\begin{array}{@{}rcl@{}} x=\frac{2 \times k}{|2-C-(\sqrt{C \times (C-4)})|}, \end{array} $$

where *k* is a random value in [0,1] and *C*=*C*_1_+*C*_2_, *C*≥4. According to Clerc [18], using a constriction coefficient results in particle convergence over time. This latter, denoted as PSO version II, updates the velocity as formalized in Eq. (2).

#### A simulated annealing approach

##### General presentation

The original Simulated Annealing (SA) method is a local search based threshold class algorithm. Basically, a threshold algorithm is a loop in which a move is either done or not, according to a given criterion and until reaching a freeze [19]. Specifically, after an initialization step, this loop is composed by (a) a move in the neighborhood of the current solution, (b) an evaluation of this new position by a real-valued scoring function, then (c) a test, given a well chosen criterion, to store this position as the new best one. Various criteria can be considered. For instance, if a position is evaluated as a better solution than the best existing one, it becomes the reference solution for next iterations when the acceptation criterion is “only if best cost (score)” algorithm, which is a variant of a classical greedy local search [20]. The “all is accepted” algorithm produces, for its part, a random walk. Finally, between these two extremes situations, an acceptation criterion allows to store sometimes too positions with poorer scores than the best solution, which is an upward move via a stochastic component to avoid local minima. Such a stochastic approach facilitates theoretical analysis of asymptotic convergence. As such algorithms can be successfully used for a broad range of optimization problems, SA has been largely covered in the literature during the last decades [20, 21], for both empirical [22, 23] – typically on NP-hard problems – and theoretical perspectives [20, 24].

In simulated annealing, the criterion is inspired by the Metropolis-Hastings statistical (Markov chain Monte Carlo) thermodynamics algorithm [20]. SA simulates the cooling of a material in a heat bath until a steady (frozen or thermodynamic equilibrium) state. When the solid material is heated over its melting point, its solidification rate induces its structural properties. Two major antagonistic strategies are commonly used. On the one hand, after a fast cooling (quenching), the steady state is constituted by different thermodynamic free level areas. This corresponds to a local minimum for a local search, when considering energy as a score. On the other hand, after a slow cooling (annealing), almost one sole thermostatic level is expected, which corresponds to a global minimum. As feasible solutions of SA are system states, the structural proximity of the latter leads to the concept of solution neighborhood.

Thermodynamic laws show that at temperature *t*, the probability to increase in energy of the value *δE* is given by *p*(*δE*)= exp(−*δE*/*kt*) with *k* equal to the Boltzmann’s constant. Metropolis simulations [25] consist in the generation of a state perturbation, in the evaluation of energy modification, and finally in the decision to reject or not the new state according to the probability *p*(*δE*). That is, the probability to keep a better (lower) level of energy is 1, while the one to keep an infinitely worst level of energy is equal to 0. Or, in other words, the likelihood to save a given state decreases as the energy level increases. A criterion to increase the probability to reach convergence is the so-called logarithmic fading of control parameter (i.e., temperature). The simplest choice is *t*_*n*+1_=*C*·*t*_*n*_, where *C*∈[0,1] is a constant. A best global solution is reached by searching series of equilibria. Each equilibrium is obtained by series of Metropolis thresholds. The stop condition is typically an arbitrary duration or a number of loop iterations. Then the temperature is decreased and the last obtained equilibrium becomes the starting state for a new series of thresholds. The final stop is triggered if no improvement has been found since an arbitrary number of equilibria. Let us finally notice that, as a large set of temperature cooling schedules (decreasing function [26, 27]), of moving functions, of criteria, of strategies regarding initial values, of improvements on score function, of stop criteria, and even of theoretical modeling [20, 28–30] have been proposed in the literature [29, 31, 32], simulated annealing should be regarded more as a large family of algorithms than as a single one. Some members of the family including Basin Hopping [33] are themselves described as frameworks for ad-hoc global optimization algorithms.

**Fig. 2:**
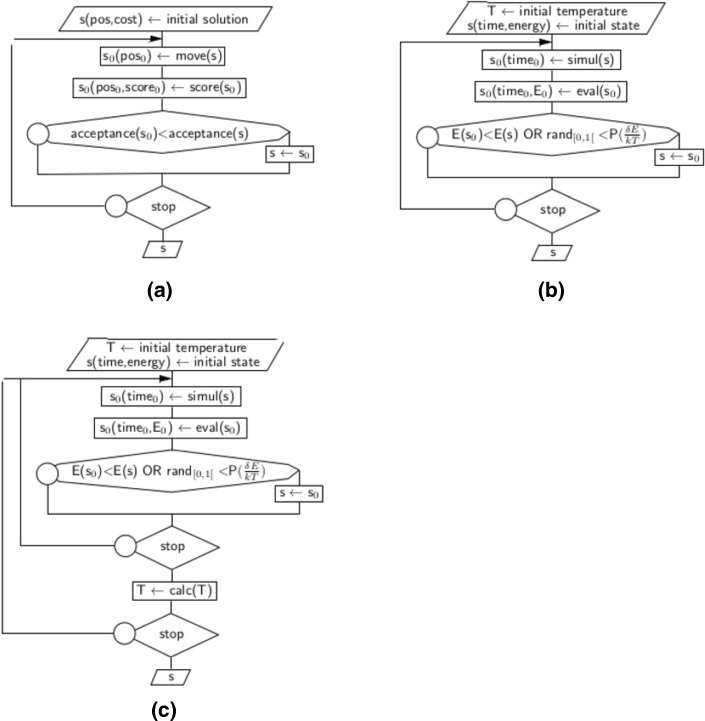
Simulated annealing as a threshold class algorithm. **a** Generic threshold class algorithm. **b** Metropolis algorithm. **c** Simulated annealing algorithm

A general overview of our proposal can be found in Fig. 2, while algorithm details are provided hereafter.

##### Designing SA for phylogenetic studies

The objective is now to apply the simulated annealing method to find the largest subset of core genes that leads to the most supported phylogenetic tree. Intermediate computations of subsets will help to understand, using regressions, the effects of given genes on both topology and supports. However, SA is complex to set up in practice, and finding new optima in finite time cannot be guaranteed, as reported by Aarts, Korst, and van Laarhoven [19]. To enlarge the probability of success, we targeted the following requirements during our experiments:

- a cooling schedule fitting with complexity, time, convergence, and precision considerations (cf. the temperature scheduling paragraph below);
- a concise representation for the problem under consideration (detailed in “About a relevant configuration of SA according to the state space”);
- a moving function adapted to the state solution space (same paragraph as above);
- and, similarly, an acceptation function adapted to the state space (see the “Proposed SA optimization”).

These four requirements are discussed hereafter.

##### Temperature scheduling.

A criterion to increase the probability to reach convergence is the so-called logarithmic fading of control parameter (*i.e.*, temperature). The most simple choice is $t_{n+1}= C\dot t_{n}$, where *C*∈[0,1] is a constant. However, according to our experiments, such a solution is not able to produce relevant results in the phylogenetic problem under consideration. This is why the control parameter has been updated following a tiered approach, leading to an inhomogeneous Markov model: the temperature decreases only after the end of its associated Markov chain. Additionally, near an equilibrium, the Markov chain length must increase when the control parameter decreases. But, as above, at low temperature the computation time may become prohibitive without any synchronisation between the control parameter and the Markov chain characteristics. To solve such an issue, various schedule solutions proposed in the literature link these two parameters. After having tested classical benchmarking functions like the well known three-hump camel, Levi, and Booth, we finally have chosen:

$$t_{n+1}=\left(\frac{t_{f}}{{t_{i}^{\frac{1}{n_{m}-1}}}}\right)\times t_{n}$$

where *t* is the control parameter, *t*_*i*_ and *t*_*f*_ are respectively the maximum (initial) and minimum (final) of allowed control parameter values for the SA computation, while *n*_*m*_ is the maximal number of Markov chains (equal to the temperature steps) allowed during computation.

##### About a relevant configuration of SA according to the state space.

As in the other methods, the state space is constituted by Boolean vectors *X*_*i*_ of the form (*X*_*i*1_,…,*X*_*in*_), where *n* is the number of core genes. *X*_*ij*_ is equal to 1 if and only if gene number *j* in alphabetic order is in the alignment provided to the phylogenetic tool. We thus navigate again on the *n*-cube on which each node (that is, each state) corresponds to a subset of core genes and has additionally a labeled value provided by the subset scoring function which is again the average between the lowest bootstrap and the number of selected core genes. We can easily define a distance between two points inside this cube, like an Hamming distance between Boolean vectors, and the node score can be considered as the altitude of the current position.

To sum up, there is a topology on the state space, with neighborhood notion between two states, while the altitude (the score of a subset of genes, which is related to the SA energy) is varying between two locations. Both the density and the form of energy peaks are varying through the landscape. Neighborhoods and moves, acceptation probability, temperature scheduling functions, and their related initial values are dependant on the characteristics, or the topology, of this state space. Obviously, there is no general way to set up the parameters of the simulated annealing in this situation, as usually with such heuristics. Even choosing close configurations of closely related problems like similar chloroplasts is not a guarantee of success.

Having these considerations in mind, we have stated some hypotheses at the basis of the neighboring notion. First of all, we assume that a solution is better if it is closer to the whole core genome, so improving the number of 1’s in the Boolean vector is a desired trend. Secondly, we assume no correlation between genes, and so removing (or adding) one gene cannot modify so much the scoring function. As a consequence, the next investigated state should be near the previous one, in terms of Hamming distance, and most likely with a similar or larger number of active genes. In particular, moves in the state space cannot be randomized as what occurs in the original SA algorithm. Furthermore, the starting state must be the Boolean vector constituted by 1’s (that is, the whole core genome), while the scoring function must preferably tend to add genes in the considered subset (if possible). With such requirements, the neighborhood function has been designed as follows:

- A number between 1 and *move*_*distance*_*max*_ (a parameter to set) is randomly chosen, following a Gaussian law. It corresponds to the number of coordinates that may possibly change.
- A subset of distinct coordinates are chosen accordingly, defining this move.
- For each Boolean coordinate, if the associated gene is inactive (0), it is activated (1). Otherwise, the gene is inactivated with a probability equal to $\frac {nz}{nc} \times \alpha $, where *nz* is the number of inactivated genes in the best current solution, *nc* is the total number of core genes in the problem, and *α* is a user-defined parameter.

###### Proposed SA optimization.

Scores in this proposal are obtained using RAxML [3]. As an inference of a bootstrapped and rooted phylogenetic tree may take times, and as we need to compute several trees, each calculated state is tagged so that it is never recomputed without an explicit user demand. Associated and detailed results are buffered on disk. Then a simple, reliable, and not really space-characteristics dependent solution is the synchronization of some SAs after the end of a Markov chain [34]. In order to do so, a batch of SAs is launched with the same configuration. After a chain, each running SA shares its own best known solution to a server. Then, it demands to this server if a better state has been found before starting the next chain. Finally, each SA halts after *n* local non optimizing chains. So a stopped SA is not restarted, even if a better solution is found elsewhere (*i.e.*, the proposed SA stops as soon as possible).

Acceptance function is also selected to take advantage of previous moves, to allow some (not too large) jumps. This is an adaptation of the so-called Tsallis acceptance probabilities [31] with a control parameter normalization:

$$\left(1 -\frac{(1-q)*\Delta}{\bar{\Delta}*t}\right)^{\frac{1}{1-q}},$$

where *Δ* is the score difference between the previous and current states, $\bar {\Delta }$ their mean, *t* is a control parameter, and *q* is a user-defined factor.

###### How to stop the SA.

To fix a predefined control (temperature) value needs to know some state space characteristics, so we choose an end criterion related to the absence of progression in scores. In other words, the proposed simulated annealing algorithm stops after *n* consecutive Markov chains without any score improvement. As SA is very slow on low temperatures, the choice has been to choose a small value for *n*. Then, a greedy local search can be launched on SA best states.

## Results

### Data generation

#### Genomes recovery and annotations

Seven hundred eighty complete genomes of chloroplasts have been downloaded from the NCBI, constituting the set of all available complete chloroplastic genomes at the date of the beginning of our study [8]. Various gene prediction methods have been previously tested, in order to translate these complete genomes in lists of annotated coding sequences. These methods encompass the single use of NCBI annotated genomes, the use of automatic annotation tools specific to organelles like DOGMA [1], and the mix of both.

Indeed, annotations from NCBI website are of very variable quality: humanly well-curated genomes go together with genomes having a lot of annotation errors, concerning either the gene names (classification or spelling errors) or DNA sequences (start and stop position, length). As the number of well annotated genomes was not enough to constitute a testing set for our experiments, we are then left to find an acceptable way to annotate the whole 780 complete genomes. As stated above, we tested various ways to annotate the genomes, and we evaluated them by checking their ability to recover the annotations (sequence positions and gene names) of the subset of humanly, well-curated genomes.

According to our experiments, there was no way to improve enough the quality of NCBI annotations [35]. Neither by cross-validating them using automatic annotation tools, nor by trying to correct errors in gene names and positions with these tools and some edit distances [36, 37]. Furthermore, to cluster the whole NCBI DNA sequences fail in separating well annotated genes in well separated clusters, due to junk DNA in the NCBI sequences. The large number of obvious errors in the NCBI annotated complete chloroplastic genomes can be explained by the large variety of annotation tools used during sequence submission, most of them being not specific to this kind of genomes (unlike DOGMA), to a misuse of these tools, or due to errors in manual annotations. The absence of a clear norm in the gene naming process adds difficulties, so that the sole method to provide accurate annotations to these 780 complete genomes was to constitute a basis of knowledge, with a subset of well curated genomes that represent well the plant diversity. And, to blast each genome against the basis, which is indeed what is done by DOGMA.

We finally have written a script that automatically send requests to the DOGMA web service, and recovers the annotated genomes. Due to this automatic process, the gene name spelling issue is resolved, and we can recover the clusters of homologous coding sequences by simply considering gene names. By applying the same tool for coding sequence prediction and naming process, we have resolved the problem of quality variability in annotations. And as DOGMA has been specifically designed for chloroplasts, errors in sequence positions have been reduced as possible. At this stage, and using our script on DOGMA web service, we have then a collection of 780 complete and “well” annotated chloroplastic genomes, from which gene names can be used to recover core and pan genomes of any subset of genomes.

#### Extracting subsets of genomes for simulations

To test the ability, for the three proposed metaheuristics methods, to find the largest subset of core genes that leads to the most supported trees, we needed to extract, from the set of annotated genomes, various distinct subsets that are such that:

- Using the whole core genome in the alignment, we cannot obtain a well supported tree.
- The time to compute this tree is reasonable, as we want to compute a lot of trees using a lot of subsets of core genes. For a given subset of core genes, this computation time encompasses:
  1. the multi-alignment of each core gene using Muscle [11],
  2. the concatenation of each aligned sequence to reconstruct the “sub” genome of each considered species (*i.e.*, the part corresponding to the considered subset of core genes),
  3. the computation of the best phylogenetic tree corresponding to this alignment (with RAxML [3]),
  4. the addition of bootstrap supports to this best tree using RAxML again,
  5. and finally the verification that one of these supports is lower than 95 at least. If so, this tree is considered as not well supported.

Given a subset of genomes, the multi-alignment of each core gene can be computed only once, prior to the research of the best subset of core genes leading to the most supported tree. So we do not have to consider the alignment stage when searching subsets of genomes with: (a) problematic phylogenies and (b) a time to infer their tree as low as possible. We stopped the process above before Stage 4 and we randomly pick another subset of species if the time to find their best phylogenetic trees using their whole core genome (*i.e.*, Stage 3) exceeds 10 seconds. If this computation time is below this threshold, we then compute 50 bootstraps and we check if the best bootstrapped tree has a problem of supports. If so, we have found a convenient subset of annotated genomes, on which we can test the three metaheuristics.

#### A simple comparison in small dimensions

**Fig. 3:**
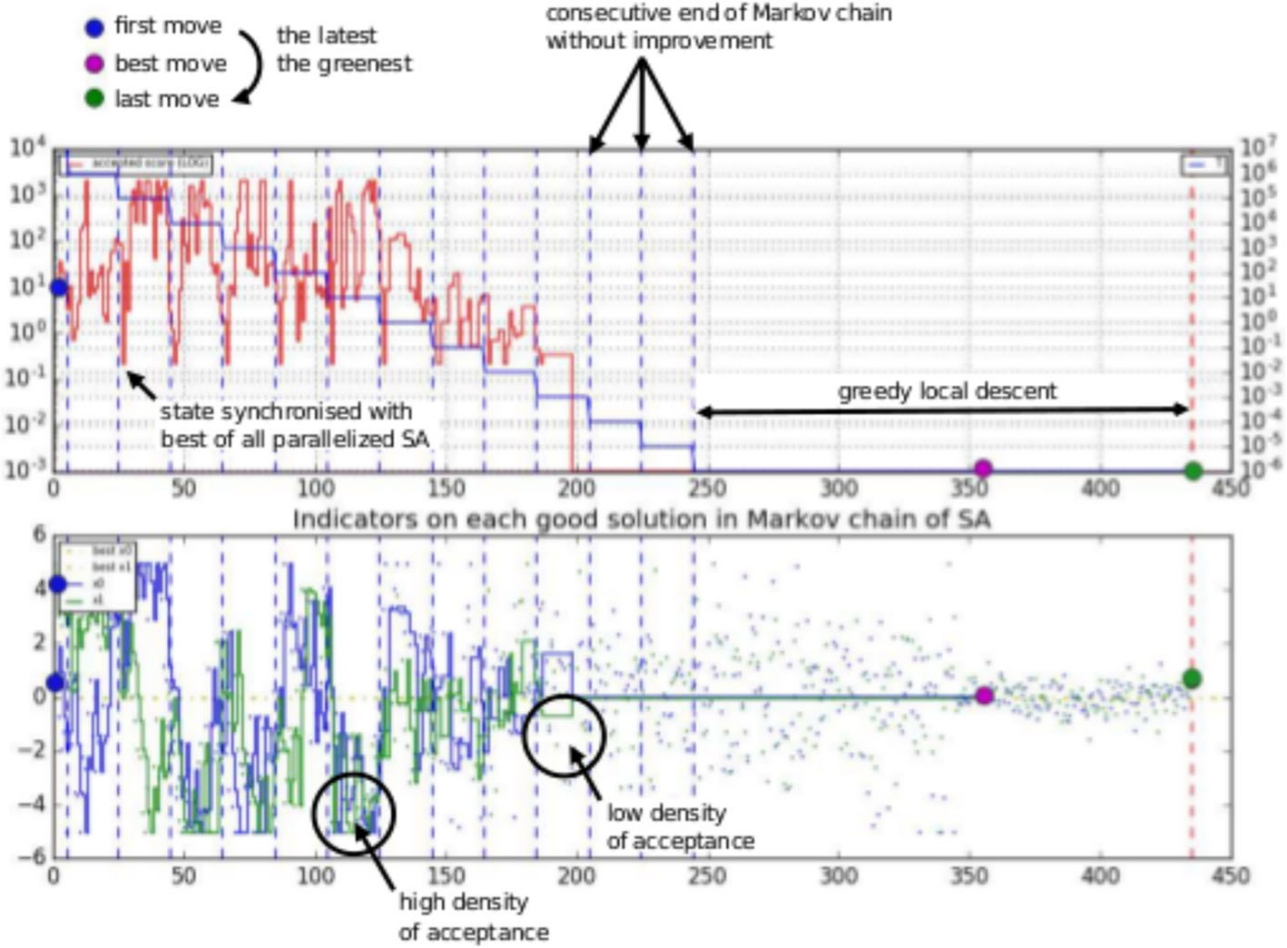
Illustration of output provided by simulated annealing approach: three-hump camel function, one instance of paralleled SA with final greedy local descent (iteration time on the abscissa)

After having executed the three metaheuristics previously described, we have validated them on test examples. We have first performed a 1D/2D comparison of the three proposals, to obtain an easy-to-understand representation of the convergence of the optimization algorithms. Figure 3 represents the output evolution of the simulated annealing, with the consecutive ends of the Markov chains and the evolution of acceptation density. From the results, we can deduce that the desired convergence behavior is well obtained, and that the comparison seems fair: no algorithm seems to underperform the other ones, and the general evolution of the energy seems to be comparable for the three algorithms. Such results allow us to further investigate simulated annealing, particle swarm optimization, and genetic algorithm for their ability to find the largest subset of core genes that leads to the most supported tree.

### Experimenting the heuristics on small collections of genomes

We first focus on small sets of species with unresolved phylogenies, for computational reasons and because small trees are easier to compare. Even in such small sets, as the core genome contains more than 100 genes, the number of combinations to test is far from what is tractable using a brute force approach. We will see that it is easy to obtain various opposed but very well supported trees using large subsets of core genes, leading to the necessity to optimize both parameters.

#### A first family of algae

We have first considered the family listed in Table 2. The detailed taxonomy information is provided hereafter.

- **Cylindrotheca closterium.** Stramenopiles; Bacillariophyta; Bacillariophyceae; Bacillariophycidae; Bacillariales; Bacillariaceae.

**Table 2:** Family number 1 (Pelargonium cotyledonis as outgroup)

Accession Nb	Name	Nb. of genes	Length (nuc.)
GenBank:[NC_024082.1]	Cylindrotheca closterium	257	165,809
GenBank:[NC_014808.1]	Thalassiosira oceanica CCMP1005	138	141,790
GenBank:[NC_025313.1]	Cerataulina daemon	195	120,144
GenBank:[NC_028052.1]	Pelargonium cotyledonis	271	166,111
GenBank:[NC_015403.1]	Fistulifera solaris	192	134,918
GenBank:[NC_024084.1]	Leptocylindrus danicus	155	125,213
- **Thalassiosira oceanica CCMP1005.** Stramenopiles; Bacillariophyta; Coscinodiscophyceae; Thalassiosirophycidae; Thalassiosirales; Thalassiosiraceae.
- **Cerataulina daemon.** Stramenopiles; Bacillariophyta; Mediophyceae; Biddulphiophycidae; Hemiaulales; Hemiaulaceae.
- **Pelargonium cotyledonis.** Viridiplantae; Streptophyta; Embryophyta; Tracheophyta; Spermatophyta; Magnoliophyta; Eudicotyledons; Gunneridae; Pentapetalae; Rosids; Malvids; Geraniales; Geraniaceae.
- **Fistulifera solaris.** Stramenopiles; Bacillariophyta; Bacillariophyceae; Bacillariophycidae; Naviculales; Naviculaceae.
- **Leptocylindrus danicus.** Stramenopiles; Bacillariophyta; Coscinodiscophyceae; Chaetocerotophycidae; Leptocylindrales; Leptocylindraceae.

**Fig. 4:**
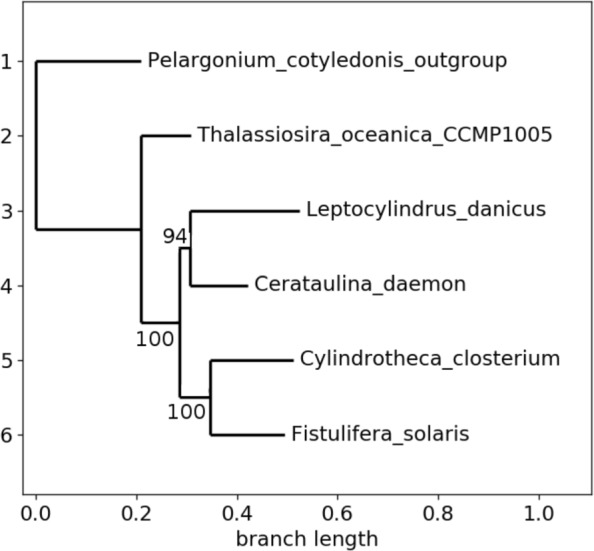
Phylogeny of family Number 1 with the whole core genome

This family is constituted by 6 genomes, of length ranging from 120,144 to 166,111 nucleotides. The number of detected genes, for its part, ranges from 138 to 271, with a core genome of 122. The phylogeny with the alignment of these core genes leads to a small weakness in one branch (bootstrap of 94), as depicted in Fig. 4. Indeed, inside this *bacillariophyta* phylum (eukaryotic algae), *C.closterium*, and *F.solaris* are naturally in the same clade, being both in the same class of *bacillariophyceae*, while the three other species are in three different classes inside this phylum.

**Fig. 5:**
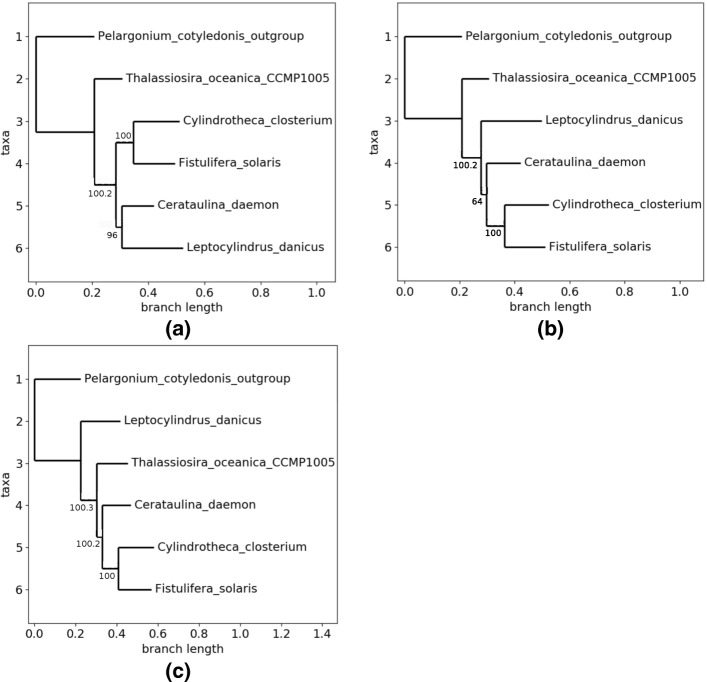
Obtained topologies with the first family. **a** Topology 0. **b** Topology 1. **c** Topology 2
- Topology 1, for its part (see Fig. 5b), has occurred twice, with a non supported branch of 64 in its best tree.

To wonder whether some genes may be responsible of such weak uncertainty, we have firstly launched the genetic algorithm: its systematic mode (in population initialization stage) indeed first tries to remove each core gene separately. This GA has stopped after 29 iterations, in systematic mode, leading to 2 topologies:

- Topology 0, depicted in Fig. 5a, has occurred 27 times. The best obtained tree has a lowest bootstrap of 96, while in average the lowest bootstrap is equal to 86.

As during these experiments, we have not leaved the initialization phase, it is useless to detail here the parameters set to configure the GA. The PSO, for its part, has been configured as follows: 3 particles, a fitness lower than 0.05 to freeze the runs, and all constants that define the velocity equal to 1. This heuristics has rapidly found a first well supported phylogenetic tree in a third different topology, and with all supports equal to 100, see Fig. 5c. However, the PSO has used only 47.5% of the core genes to reach such a tree. According to our stop criterion, this tree has not been returned by the algorithm. Indeed, this example illustrates the ability of the particle swarm optimization algorithm to more globally visit the whole space at the beginning, in order to discover regions of interest.

If we compare for instance the behavior of the PSO during the same time than the one required to finish the GA (29 iterations), we discovered 5 topologies, two of them having all their supports equal to 100 (Topologies 0 and 2 in Fig. 5, occurring respectively 17 and 7 times). They however used only between 44.26% and 48.36% at this starting point in the PSO. Bit by bit, over iterations, the percentage of core genes is enlarging, and the swarms tend to prefer the Topology 0. Finally, after 350 computed trees (which was the stopping condition), this topology has been obtained in 53.42% of the cases, and its best tree has a lowest bootstrap of 100 using 66.39% of core genes. The number of occurrences of the other topologies has growth more slowly and, even if all the bootstraps of their best representatives exceed the value of 98, the latter fails in the attempt to significantly increase the number of considered core genes in these representatives (always lower than 55.8%).

The simulated annealing, for its part, raised 3 topologies, exactly the ones depicted in Fig. 5. It has been launched with an initial temperature equal to 100, a final one of 1e-10, and an optimal exponential temperature function. Acceptation function was the Tsallis normalized one, with a *q* factor of 0.25, and initial (resp. final) acceptance of 0.7 (resp. 1e-05). A remarkable element is that these 3 topologies have the whole bootstraps equal to 100. Furthermore, Topology 2 appears as the best one according to the produced result (it was Topology 0 according to the GA, while PSO has not succeeded in separating these two topologies). With details, the SA has stopped after 364 computed trees with 6 occurrences of Topology 0, 43 of Topo. 1, and 315 for the Topology 2. Similarly, the percentage of core genes leading to the best representative in each topology is respectively of 56.56% (Topo. 0), 74.59% (Topo. 1), and 94.98% (Topo. 2), which thus outperforms the other ones according to these simulations.

Obviously, both PSO and SA have converged to local minima that are not global ones if we consider that both minimum bootstraps and proportion of core genes must be maximized. Launching them again with other initial values and parameters may select other optimal positions in the cube. The genetic algorithm with this family is emblematic, as during its initial population generation it has returned Topology 0 that is totally supported with 99.18% of the core genome. This topology seems to be an acceptable representation of the phylogenetic relationship between these chloroplasts. But it is remarkable that, using the same large proportion of core gene, we can break in the sister relationship between *L.danicus* and *C.daemon*. Indeed, this behavior has been obtained frequently with various collections of data, which will be illustrated below.

Up to now, we only have considered one problematic bootstrap, which may be easy to resolve when removing genes. New difficulties are added when there are at least two problems in the list of bootstraps, as improving the first one may lead to a decrease in the second value. We have investigated this point in the second tested family.

#### A second family with two problematic bootstraps

**Fig. 6:**
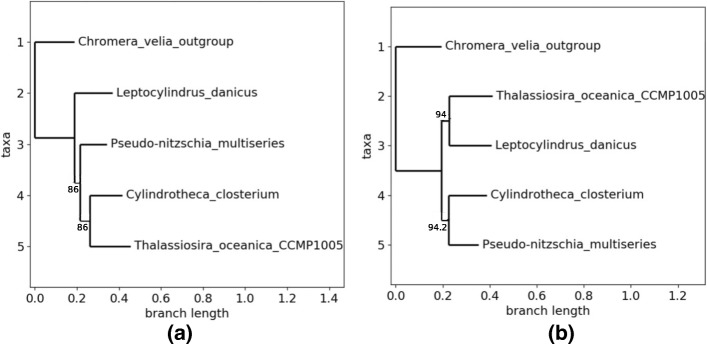
Obtained topologies with the second family. **a** Topology 0 with the whole core genome. **b** Topology 1 obtained by GA

The second small set of genomes is constituted by 4 *Bacillariophyta* plus an *Alveolata* as outgroup, as listed in Table 3. Taxonomic details are provided hereafter, while the phylogenetic tree based on the alignment of the core genome is provided in Fig. 6.

- **Cylindrotheca closterium.** Stramenopiles; Bacillariophyta; Bacillariophyceae; Bacillariophycidae; Bacillariales; Bacillariaceae.

**Table 3:** Family number 2 (Chromera velia as outgroup)

Accession Nb	Name	Nb. of genes	Length (nuc.)
GenBank:[NC_024082.1]	Cylindrotheca closterium	257	165,809
GenBank:[NC_014808.1]	Thalassiosira oceanica CCMP1005	138	141,790
GenBank:[NC_027721.1]	Pseudo-nitzschia multiseries	267	111,539
GenBank:[NC_024084.1]	Leptocylindrus danicus	155	125,213
GenBank:[NC_014340.2]	Chromera velia	265	120,426
- **Thalassiosira oceanica CCMP1005.** Stramenopiles; Bacillariophyta; Coscinodiscophyceae; Thalassiosirophycidae; Thalassiosirales; Thalassiosiraceae.
- **Pseudo-nitzschia multiseries.** Stramenopiles; Bacillariophyta; Bacillariophyceae; Bacillariophycidae; Bacillariales; Bacillariaceae.
- **Leptocylindrus danicus.** Stramenopiles; Bacillariophyta; Coscinodiscophyceae; Chaetocerotophycidae; Leptocylindrales; Leptocylindraceae.
- **Chromera velia.** Alveolata; Chromerida

The phylogenetic tree is not well-supported, having two bootstrap values of 86. Furthermore, *T.oceanica* and *L.danicus* are not sisters in this tree, while they belong in the *Coscinodiscophyceae* class of *diatom*. More seriously, the two other species belong to the *Bacillariaceae* family, which is in contradiction with this tree. It is not a necessity to recover exactly the known taxonomy, as we focus on chloroplasts, but this tree is at least suspicious if we consider both supports and taxonomy. This example illustrates the fact that to use the largest common subset of sequences is not sufficient enough to guarantee a well conducted phylogenetic study. Conversely, and obviously, to have good supports is not enough, as all best trees in the different topologies of the previous family are well supported in the SA case: the largest number of core genes must be thus coupled with the research of the best supports.

Once again, the genetic algorithm has stopped rapidly, in the systematic mode. The 22 first genes have been tested (*i.e.*, removed) before finding Topology 0 of Fig. 6a with a lowest bootstrap equal to 96 (and 99.18% of the genes), thus stopping the GA, while a new topology (Topology 1, see Fig. 6b) has occurred three times (best tree having twice 94 as bootstraps). Compared with the first family, the genetic algorithm stops here before succeeding to reinforce the confidence put in Topology 0, which justifies to test the two other approaches.

PSO heuristics produces the same two topologies after 1165 computed trees, with all supports equal to 100, and approximately the same number of trees (632 for Topo. 0 and 533 for Topo. 1) and of genes (70.49% versus 74.59%). We stopped the swarm manually, as these two scores have not been improved during the last 500 iterations. Obviously, the 3 particles have been blocked in two local extrema, and the way we configured their velocity (0.9 and 0.8 for *ϕ*_1_ and *ϕ*_2_) does not allow them to leave these optima. So we still cannot choose definitively the topology number 0.

Finally, the simulated annealing has produced 400 trees before convergence. They all belong to the two topologies detailed above. However, produced results show that Topology number 1 must be preferred, according to the SA, and this latter is neither the one obtained with the whole core genome, nor the best one according to GA. Indeed, after convergence, all bootstraps here are equal to 100 in the best tree found inside each topology. But topology of Fig. 6b has been obtained in 88.5% of the cases. More significantly, best tree in Topology 1 is obtained using 96.72% of the core genome, while for Topology 0, the best tree uses 90.98% of it. Remark that using the nine-tenths of the core genome, you can obtain a first topology with all supports equal to 100, while using more than 96% you can find a different topology with again all supports equal to 100. And, if we consider the average between the lowest bootstrap and the proportion of core genes as a score, the best topology according to GA has a score of 97.59/100, while it is of 98.36 for Topology 1 found by the SA.

## Discussion

We will now further investigate the simulated annealing convergence process, before studying more deeply the two other algorithms in a next section.

### Early analysis on SA computed problem: an illustration

**Fig. 7:**
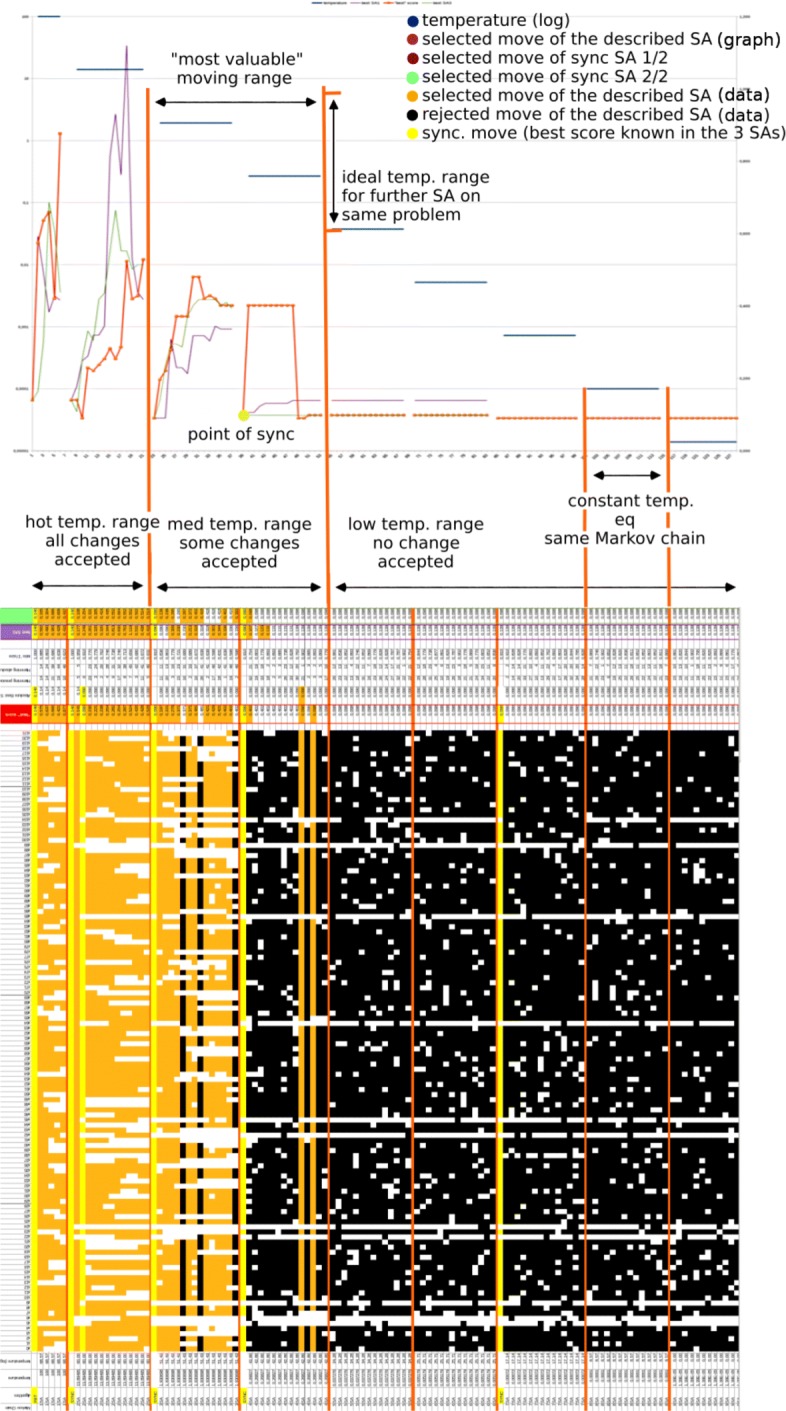
Illustration of clade analysis with a 3-parallelized SA

An example of a SA batch run (three clients on the first family described previously) is depicted in Fig. 7. For easy understanding, only some outputs have been reported in the figure.

On the lower part, all moves of the simulated annealing are reported with their nature : synchronized move in yellow (*i.e.*, copy, from a shared memory, of the best known solution found in the three SAs), move with an accepted status in orange, and rejected moves in black. Active genes are filled squares and not selected ones are white squares. Other important data for analysis are reported, such as: temperature, accepted score of other SAs (green and purple), and Hamming distance between two consecutive positions (moving behavior indicator).

On the upper part, a graph of accepted scores from the three SAs is provided, with the temperature variations due to move iterations (a lower score is a better one). As we represented the first run on a new collection of genomes, no previous configurations were available to set up the parameters. Consequently, a broad range of temperatures has been considered. The Markov chains are short, in order to reduce the computation time. From this beginning of an experiment, it can be deduced that:

- the temperature ranges well, allowing further experiments on the same set of data;
- even with a poor configuration, SAs have found a score “not so bad”, which is associated to a topology that other heuristics have considered as a good one.

**Fig. 8:**
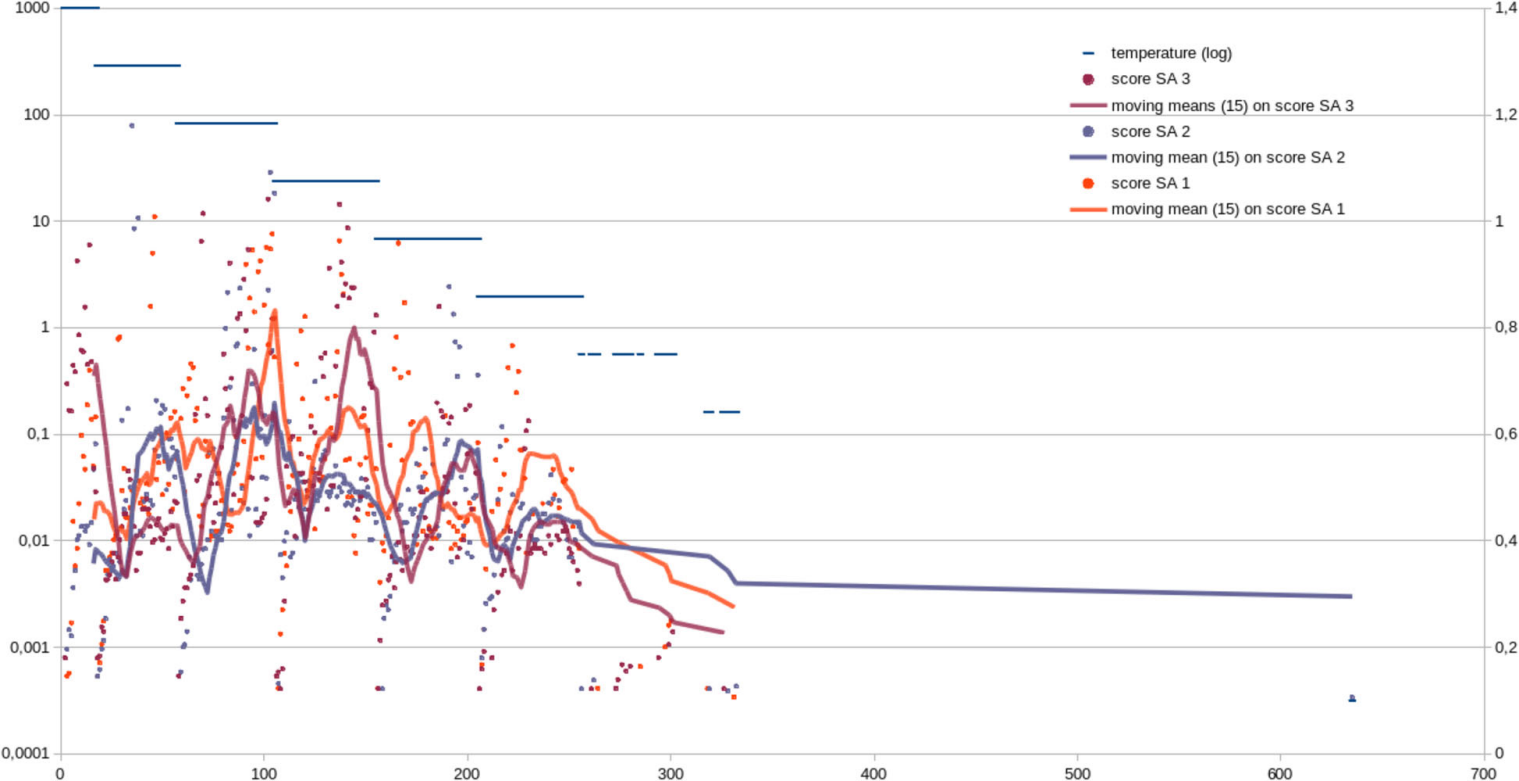
Illustration of convergence on 3-parallelized SA

Another SA evolution is provided in Fig. 8, in which the three main curves do not represent moves, but “moves of locally selected moves”, which are stabilized over time.

### A further comparison of the distributed versions of GA and BPSO performance

During the experiments of the previous section, it was impossible to evaluate in practice the behavior of the genetic algorithm, as this latter found an optimum during the initialization stage. Similarly, BPSO has underperformed the two other algorithms, while SA always produced interesting results. This is why we decided, after having studied the SA evolution on the first family, to further investigate both BPSO (with its two velocity versions) and GA in large collections of experiments, distributed in a supercomputer facilities. To do so, 12 groups of plant genomes have been extracted from our set of annotated genomes. They have been applied on our two swarm versions, and results have been compared to the genetic algorithm ones.

Comparisons are provided in Tables 4 and 5. In these tables, *Topo.* column stands for the number of topologies, *NbTrees* is the total number of obtained trees using 10 swarms, *b* is the minimum bootstrap value of selected *w*, 100−*p* is the number of missing genes in *w* and *Occ.* is the number of occurrences of the best obtained topology from 10 swarms. As can be seen in these tables, the two versions of BPSO did not provide the same kind of results:

- In the case of *Chlorophyta*, *Pinus*, and *Bambusoideae*, the second version of the BPSO has outperformed the first one, as the minimum bootstrap *b* of the best tree is finally larger for at least one swarm.

**Table 4:** Groups from BPSO version I

Group	Topo.	NbTrees	*b*	|*c*|	100−*p*^′^	Occ.	Swarms	Particles
*Pinus*	3	508	98	79	32	462	1,2,3,4,5,6,7,8,9,10	10
*Pinus*	3	530	94	79	11	129	1,2,3,4,5,6,7,8,9,10	15
*Picea*	1	100	100	85	42	100	1,2,3,4,5,6,7,8,9,10	10
*Picea*	1	428	100	85	13	428	1,2,3,4,5,6,7,8,9,10	15
*Magnoliidae*	3	750	100	79	20	613	1,2,3,4,5,6,7,8,9,10	10
*Magnoliidae*	3	845	100	79	19	707	1,2,3,4,5,6,7,8,9,10	15
*Ericales*	30	344	53	84	26	185	1,2,3,4,5,6,7,8,9,10	10
*Ericales*	34	555	54	84	5	363	1,2,3,4,5,6,7,8,9,10	15
*Bambusoideae*	8	496	72	94	37	456	1,2,3,4,5,6,7,8,9,10	10
*Bambusoideae*	11	694	69	94	18	621	1,2,3,4,5,6,7,8,9,10	15
*Eucalyptus*	16	828	86	83	7	632	1,2,3,4,5,6,7,8,9,10	10
*Eucalyptus*	20	1073	86	80	4	845	1,2,3,4,5,6,7,8,9,10	15
*Malpighiales*	34	327	65	78	35	233	1,2,3,4,5,6,7,8,9,10	10
*Malpighiales*	38	483	69	78	40	326	1,2,3,4,5,6,7,8,9,10	15
*Chlorophyta*	25	191	70	24	11	109	1,2,3,4,5,6,7,8,9,10	10
*Chlorophyta*	29	94	68	24	11	1	1,2,3,4,5,6,7,8,9,10	15
*Euglenozoa*	3	450	100	26	7	292	1,2,3,4,5,6,7,8,9,10	10
*Euglenozoa*	3	520	100	26	4	491	1,2,3,4,5,6,7,8,9,10	15
*Ehrhartoideae*	2	23	100	81	0	23	1,2,3,4,5,6,7,8,9,10	10
*Ehrhartoideae*	3	455	100	81	0	451	1,2,3,4,5,6,7,8,9,10	15
*Trebouxiophyceae*	3	409	100	41	2	405	1,2,3,4,5,6,7,8,9,10	10
*Trebouxiophyceae*	3	415	100	41	8	354	1,2,3,4,5,6,7,8,9,10	15
*Poeae*	1	971	100	80	9	971	1,2,3,4,5,6,7,8,9,10	10
*Poeae*	1	1399	100	80	20	1399	1,2,3,4,5,6,7,8,9,10	15

**Table 5:** Groups from PSO version II

Group	Topo.	NbTrees	*b*	|*c*|	100−*p*^′^	Occ.	Swarms	Particles
*Pinus*	3	615	98	79	14	275	1,2,3,4,5,6,7,8,9,10	10
*Pinus*	3	628	100	79	12	558	1,2,3,4,5,6,7,8,9,10	15
*Picea*	1	635	100	85	14	635	1,2,3,4,5,6,7,8,9,10	10
*Picea*	1	821	100	85	15	821	1,2,3,4,5,6,7,8,9,10	15
*Magnoliidae*	3	494	100	79	16	73	1,2,3,4,5,6,7,8,9,10	10
*Magnoliidae*	3	535	100	79	42	384	1,2,3,4,5,6,7,8,9,10	10
*Bambusoideae*	6	952	84	81	23	94	1,2,3,4,5,6,7,8,9,10	10
*Bambusoideae*	9	1450	82	81	18	113	1,2,3,4,5,6,7,8,9,10	15
*Eucalyptus*	17	972	88	80	18	618	1,2,3,4,5,6,7,8,9,10	10
*Eucalyptus*	23	1439	92	80	10	843	1,2,3,4,5,6,7,8,9,10	15
*Chlorophyta*	25	529	71	24	6	397	1,2,3,4,5,6,7,8,9,10	10
*Chlorophyta*	46	1500	82	24	11	397	1,2,3,4,5,6,7,8,9,10	10
*Ericales*	30	97	51	84	11	56	1,2,3,4,5,6,7,8,9,10	10
*Ericales*	34	1257	52	84	7	800	1,2,3,4,5,6,7,8,9,10	15
*Malpighiales*	35	725	72	79	25	445	1,2,3,4,5,6,7,8,9,10	10
*Malpighiales*	86	1464	84	79	45	359	1,2,3,4,5,6,7,8,9,10	15
*Euglenozoa*	3	197	100	26	1	165	1,2,3,4,5,6,7,8,9,10	10
*Euglenozoa*	3	450	100	26	10	393	1,2,3,4,5,6,7,8,9,10	15
*Ehrhartoideae*	1	24	100	81	10	24	1,2,3,4,5,6,7,8,9,10	10
*Ehrhartoideae*	1	20	100	81	9	20	1,2,3,4,5,6,7,8,9,10	15
*Trebouxiophyceae*	3	319	100	41	1	313	1,2,3,4,5,6,7,8,9,10	10
*Trebouxiophyceae*	3	818	100	41	2	81	1,2,3,4,5,6,7,8,9,10	15
*Poeae*	1	991	100	80	22	991	1,2,3,4,5,6,7,8,9,10	15
*Poeae*	1	1490	100	80	26	1490	1,2,3,4,5,6,7,8,9,10	15
- In the *Ericales* case, the first version has produced the best result.

**Fig. 9:**
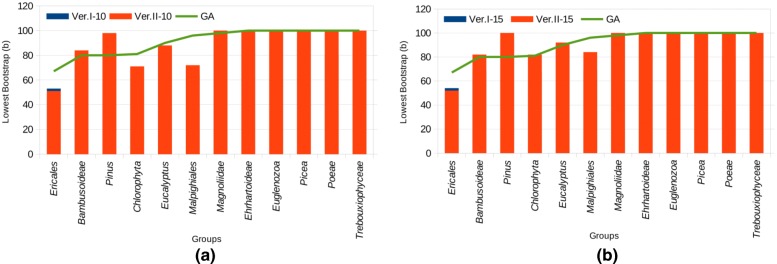
BPSO with 10 and 15 particles vs. GA. **a** BPSO with 15 particles vs. GA. **b** BPSO with 10 particles vs. GA

We can also remark that *Malpighiales* has better *b* in GA than the two versions of BPSO. *Pinus* data set has got maximum bootstrap *b* larger than what has been obtained using the genetic algorithm, while *Picea* and *Trebouxiophyceae* have got the same values of *b* than with genetic algorithm. Further comparison results between GA and both versions of BPSOs are provided in Fig. 9.

According to this figure, we can conclude that the two approaches lead to quite equivalent bootstrap values in most data sets, while on particular subgroups obtained results are complementary. In particular, BPSO often produces better bootstraps than GA (see *Magnoliidae* or on *Bambusoideae*), but with a larger number of removed genes. Finally, using 15 particles instead of 10 does not improve so much the obtained results (see Fig. 9 and Table 6).

**Table 6:** Comparison between the two versions of binary particule swarm optimization (with 10 or 15 particles) and the genetic algorithm

	BPSO version I		BPSO version II		GA
Group of species	10	15	10	15	
*Ericales*	53	54	51	52	67
*Bambusoideae*	72	69	84	82	80
*Pinus*	98	94	98	100	80
*Chlorophyta*	70	68	71	82	81
*Eucalyptus*	86	86	88	92	90
*Malpighiales*	65	69	72	84	96
*Magnoliidae*	100	100	100	100	98
*Ehrhartoideae*	100	100	100	100	100
*Euglenozoa*	100	100	100	100	100
*Picea*	94	100	100	100	100
*Poeae*	80	80	100	100	100
*Trebouxiophyceae*	100	100	100	100	100

## Conclusion

This article has presented three metaheuristics to produce a well supported phylogenetic tree based on the largest possible subset of core genes. These methods are, namely, genetic algorithm, binary particle swarm optimization, and simulated annealing. They have been evaluated on various sets of chloroplast species and deployed on a supercomputer facilities. Given the average between the percentage of core genes and the lowest bootstrap as scoring function, we have shown on simple examples that, given a set of species, various global optima with contradictory topologies can be reached. These first experiments emphasize that sometimes the phylogeny of chloroplasts cannot perfectly be resolved using a tree: a phylogenetic network may be more close to the reality, branches within this network being as strong as the associated tree topology is frequent.

Phylogenetic networks can be obtained by merging gene trees. In future work, we will propose a way to obtain such networks with large subsets of random core genes, and will show that such ways reinforce the stability and the confidence of the network. We intend to provide too criteria to decide if either a tree or a network is preferable for a given set of DNA sequences. We will measure the impact of this choice and of the coexistence of different well-supported topologies on works like ancestral genome reconstruction. Finally, the various ways to set up the metaheuristics proposed here will be systematically investigated, to find the best manner to configure these ones when targeting the largest subset of core genes leading to the most supported tree or network.

## Abbreviations

BPSO: Binary particle swarm optimization
GA: Genetic algorithm
PSO: Particle swarm optimization
SA: Simulated annealing
